# Derivation of Probability Density Function of Signal-to-Interference-Plus-Noise Ratio for the MS-to-MS Interference Analysis

**DOI:** 10.1155/2013/143970

**Published:** 2013-12-24

**Authors:** Ho-Kyung Son, Che-Young Kim

**Affiliations:** ^1^Radio Technology Research Department, Electronics and Telecommunications Research Institute, 138 Gajeongno, Yuseong-gu, Daejeon 305-700, Republic of Korea; ^2^School of the Electronics Engineering, Kyungpook National University, Sankyuk-dong, Puk-gu, Daegu 702-701, Republic of Korea

## Abstract

This paper provides an analytical derivation of the probability density function of signal-to-interference-plus-noise ratio in the scenario where mobile stations interfere with each other. This analysis considers cochannel interference and adjacent channel interference. This could also remove the need for Monte Carlo simulations when evaluating the interference effect between mobile stations. Numerical verification shows that the analytical result agrees well with a Monte Carlo simulation. Also, we applied analytical methods for evaluating the interference effect between mobile stations using adjacent frequency bands. The analytical derivation of the probability density function can be used to provide the technical criteria for sharing a frequency band.

## 1. Introduction

In wireless communication, interference between two systems occurs when these systems operate at overlapping frequencies, in the same physical environment, at the same time, with overlapping antenna patterns. In other words, these interference problems occur because several radio systems must share a limited frequency band. New technologies continue to appear, but the frequency resources are limited. Therefore, efficient use of the limited frequencies is important. To provide a more efficient use of limited frequency resources, accurate prediction of the potential interference effects between wireless communication systems is necessary.

Recently, mobile stations have populated over indoors or outdoors environments. The indoor users are mainly located inside offices, shopping malls, cafes, subways, offices, or meeting rooms. The outdoor users are typically located in squares, sidewalks, or parking spaces. Analyzing the effects of interference in these locations is necessary since these terminal stations are often in use at the same time and produce interference to each other.

So far, the various researches associated with interference analysis between terminals of Global System for Mobile Communication (GSM), Code Division Multiple Access (CDMA), Wideband Code Division Multiple Access (WCDMA), and Long Term Evolution (LTE) have been done [1–4]. Generally, the Monte Carlo method must be conducted to capture realistic system behavior and to determine the levels of interference that will be experienced within a real system when considering mobile station to mobile station interference. In [1], the researchers assume that users are uniformly located over the network area. This is not, however, the actual situation. Instead, users in the radio cell locate inside clusters. In [2], researchers derive the capacity reduction of a WCDMA downlink due to mobile-to-mobile interference, considering nonuniform user distribution. In [3], the author evaluates the impact of user equipment to user equipment interference between LTE systems operated by different operators at adjacent frequency bands, and real user distribution is also taken into account. In [4], appropriate Block Emission Mask (BEM) baseline limits are subsequently derived via Monte Carlo simulations based on interferer terminal station spatial densities commensurate with those observed in busy hotspots.

In this paper, we analytically derive the probability density function (pdf) of the signal-to-interference-plus-noise ratio (SINR) considering mobile-to-mobile interference in a wireless network without recourse to Monte Carlo simulations. We compare the analytical result with a Monte Carlo simulation to verify the analytical method and apply the analytical method for evaluating the interference effect of LTE's user equipment on a Trunked Radio Service (TRS)'s mobile station using adjacent frequency bands.

This paper is organized as follows. In Section 2, the interference model is described and we derive the pdf of SINR for mobile-to-mobile interference. In Section 3, the analytical method results are also compared with a Monte Carlo simulation, and the example of interference analysis using the derived analytical method is presented. Finally, the conclusions are discussed in Section 4.

## 2. Analytical Derivation of the Pdf

**Figure 1:**
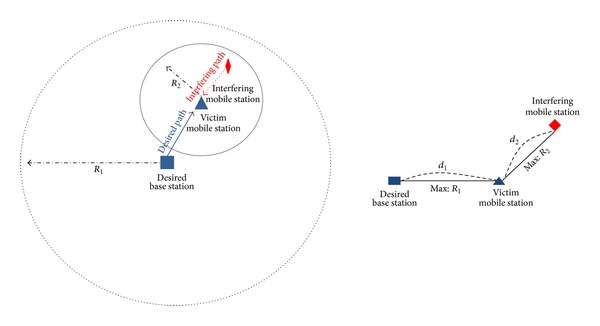
Interference model for the derivation of the pdf.

Figure 1 shows an interference model used to derive the pdf of SINR for analyzing mobile-to-mobile interference. We assume circular cells instead of hexagonal cells for simplicity, and the victim mobile station is at a random uniformly distributed location within the cell radius, *R*_1_. Then we deploy interfering mobile stations at random locations within a hotspot cell radius of the victim mobile station, *R*_2_. The pdf of SINR is calculated assuming one uniformly distributed interfering mobile station. The result obtained can be easily extended to multiple interfering mobile stations.

We also assumed that the noise power is relatively very small compared to adjacent channel interference (ACI). The ACI level is much higher than the thermal noise power level at the receiver of victim mobile station if located within the range of hotspot cell radius. The SINR in the victim mobile station can be denoted as follows:

(1) SINR=PwantedPnoise+Pinterfering=C1(d1)−α1·10PD/1010PN/10+10PI/10·10−κI/10·C2(d2)−α2≈C1C2·10(PD−PI+κI)/10·d2α2d1α1,

where *P*_*D*_ is the transmitted power of the desired base station, *P*_*I*_ is the transmitted power of the interfering mobile station, *P*_*N*_ is the receiver thermal noise power, *κ*_*I*_ is the adjacent channel interference ratio (ACIR) in decibels, *d*_1_ is the distance between the victim mobile station and the desired base station to which it is connected, *d*_2_ is the distance between the victim mobile station and the interfering mobile station, *C*_1_ is the constant, *α*_1_ is the path loss exponent for the desired path, *C*_2_ is the constant, and *α*_2_ is the path loss exponent for the interfering path.

Transforming the SINR in (1) into a dB scale and using the substitution *β* = log_10_ *e* yield

(2) SINR=(10 log10(C1C2)+PD−PI+κI)+10βln⁡d2α2d1α1=K+10·α1βln⁡1d1+10·α2βln⁡d2,

where *K* is a constant and *d*_1_ and *d*_2_ are random variables. All the mobile stations are assumed to be mutually independent and uniformly distributed in their corresponding cells. Thus, the pdf of *d*_1_ and *d*_2_ are given as [5]

(3) $$\begin{array}{c} f_{d_{i}}\left(x\right)=\{\begin{array}{cc} \frac{2}{D_{i}^{2}}x, & 0\leq x\leq R_{i}\\ 0, & \text{otherwise}. \end{array} \end{array}$$

The pdf of random variables *y*_1_ = ln⁡(1/*d*_1_) and *y*_2_ = ln⁡(*d*_2_) can be written as

(4) $$\begin{array}{c} f_{Y_{1}}\left(y_{1}\right)=\{\begin{array}{cc} f_{X_{1}}\left(x_{1}\right)\left|\frac{dx_{1}}{dy_{1}}\right|=\frac{2}{R_{1}^{2}}e^{-2y_{1}}, & -\ln\left(R_{1}\right)\leq y_{1}\leq \infty \\ 0, & \text{otherwise}, \end{array}\\ f_{Y_{2}}\left(y_{2}\right)=\{\begin{array}{cc} f_{X_{2}}\left(x_{2}\right)\left|\frac{dx_{2}}{dy_{2}}\right|=\frac{2}{R_{2}^{2}}e^{2y_{2}}, & -\infty \leq y_{2}\leq \ln\left(R_{2}\right)\\ 0, & \text{otherwise}. \end{array} \end{array}$$

And the pdf of random variables *z*_1_ = *a*_1_ln⁡(1/*d*_1_) and *z*_2_ = *a*_2_ln⁡(*d*_2_) can be written as

(5) $$\begin{array}{c} f_{Z_{1}}\left(z_{1}\right)=\{\begin{array}{cc} \frac{2}{a_{1}R_{1}^{2}}e^{-2z_{1}/a_{1}}, & -a_{1}\ln\left(R_{1}\right)\leq z_{1}\leq \infty \\ 0, & \text{otherwise}, \end{array}\\ f_{Z_{2}}\left(z_{2}\right)=\{\begin{array}{cc} \frac{2}{a_{2}R_{2}^{2}}e^{2z_{2}/a_{2}}, & -\infty \leq z_{2}\leq a_{2}\ln\left(R_{2}\right)\\ 0, & \text{otherwise}. \end{array} \end{array}$$

After calculating the pdf of each random variable, the sum of the two random variables can be obtained using the following convolution [6]:

(6) fZ(z)=∫−∞∞fX,Y(x,z−x)dx=∫−∞∞fX(x)fY(z−x)dx,

where *f*_*X*_(*x*) and *f*_*Y*_(*z* − *x*) are given by

(7) $$\begin{array}{c} f_{X}\left(x\right)=\{\begin{array}{cc} \frac{2}{a_{1}R_{1}^{2}}e^{-2x/a_{1}}, & -a_{1}\ln\left(R_{1}\right)\leq x\leq \infty \\ 0, & \text{otherwise}, \end{array}\\ f_{Y}\left(z-x\right)=\{\begin{array}{cc} \frac{2}{a_{2}R_{2}^{2}}e^{2(z-x)/a_{2}}, & z+a_{2}\ln\left(R_{2}\right)\leq x\\ 0, & \text{otherwise}. \end{array} \end{array}$$

If *z* − *a*_2_ln⁡(*R*_2_)<−*a*_1_ln⁡(*R*_1_), the pdf of SINR is

(8) fZ(z)=∫−a1ln⁡⁡(R1)∞fX(x)fY(z−x)dx=∫−a1ln⁡⁡(R1)∞4a1a2R12R22e−2x/a1+2(z−x)/a2dx=2R12a1/a2(a1+a2)R22e(2/a2)z.

Else, if *z* − *a*_2_ln⁡(*R*_2_)≥−*a*_1_ln⁡(*R*_1_), the pdf of SINR is

(9) fZ(z)=∫z−a2ln⁡⁡(R2)∞fX(x)fY(z−x)dx=∫z−a2ln⁡⁡(R2)∞4a1a2R12R22e−2x/a1+2(z−x)/a2dx=2R22a2/a1(a1+a2)R12e−(2/a1)z.

The final pdf of SINR can be written as

(10) fSINR(t)=fz(t−K)={2R12a1/a2(a1+a2)R22e(2/a2)(t−K),t<ln⁡(R2a2R1a1)+K2R22a2/a1(a1+a2)R12e−(2/a1)(t−K),t≥ln⁡(R2a2R1a1)+K.

In addition, we derive the pdf of SINR by applying the shadowing model to the desired link between the desired base station and the victim mobile station. First, the expression of the SINR in (1) applied to the shadowing model is as follows:

(11) SINR=K+ξ+10·α1βln⁡1d1+10·α2βln⁡1d2=X(SINR  without  shadowing)+ξ,

where *ξ* is a random component due to shadowing on the desired path. The shadowing model is generally assumed to be log-normal distribution, so a probability distribution is the normal distribution with a standard deviation *σ* and it can be expressed as follows [6]:

(12) $$\begin{array}{c} p\left(\xi \right)=\frac{1}{\sqrt{2\pi \sigma }}\exp\left(-\frac{\xi^{2}}{2\sigma^{2}}\right). \end{array}$$

Equation (11) becomes the sum of a random variable with the probability density function of (10) and random variables with normal distribution.

Then, using the convolution in (6), we can obtain the probability density function of the sum of two random variables. This can be written as follows:

(13) fZ(z)=2R22a2/a1e2σ2/a12(a1+a2)R12e−(2/a1)(z−K)×Q(1σ(−z+ln⁡(D2a2D1a1)+K+2σ2a1))+2R12a1/a2e2σ2/a22(a1+a2)R22e−(2/a2)(z−K)×Q(1σ(−z+ln⁡(D2a2D1a1)−K+2σ2a2)).

This pdf can be used to examine many different mobile-to-mobile interference problems in wireless communication.

## 3. Results and Discussion

We compared the proposed analytical methods and a Monte Carlo simulation in order to obtain sufficient evidence as to whether both approaches lead to similar results. The Monte Carlo method has been implemented by the way of ERC report [7].

Table 1 presents the parameters used for simulation. The 900 MHz frequency band is chosen for the simulation. The path loss model for the path between the base station and the mobile station is chosen by the Hata model [8], and the path loss model for the path between the mobile stations is selected by the Motley-Keenan formula [9]. The value *α*_1_ = 3.52, *C*_1_ = 10^−2^, *α*_2_ = 2, and *C*_2_ = 10^−3.15^ can be obtained through the two path loss models. The transmitted power of the desired base station is assumed to be 56 dBm and the transmitted power of the interfering mobile station is set to be 24 dBm. The cell radius *R*_1_ is assumed to be 2000 m.

**Figure 2:**
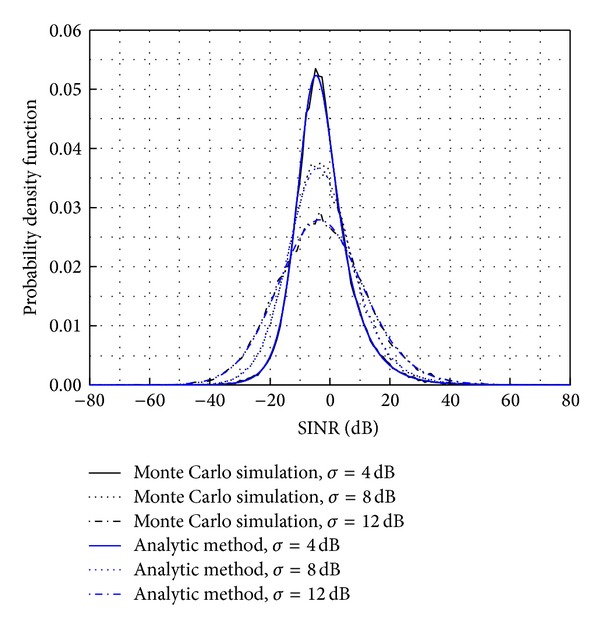
A comparison of the pdf result between the analytical method and the Monte Carlo simulation.

Figure 2 depicts three different plots depending on the standard deviation of the log-normal shadowing model *σ*. The cell radius *R*_2_ is assumed to be 50 m in this simulation. The results show that the analytical pdf is in agreement with the Monte Carlo simulation, and the mean of the SINR will not change as the standard deviation of the log-normal shadowing changes.

**Figure 3:**
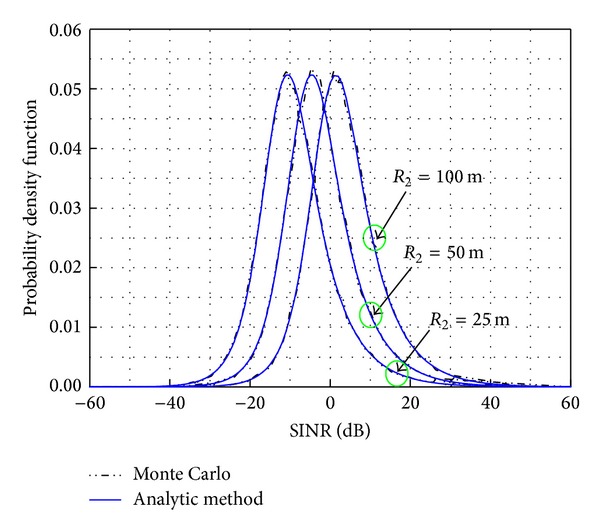
The pdf of the SINR for different values of cell radius, *R*_2_.

Figure 3 depicts three different plots depending on the hotspot cell radius. The standard deviation of log-normal shadowing model *σ* is assumed to be 4 dB. As the cell radius, *R*_2_, increases, the average received interfering signal power decreases and it leads to a higher SINR mean value.

**Figure 4:**
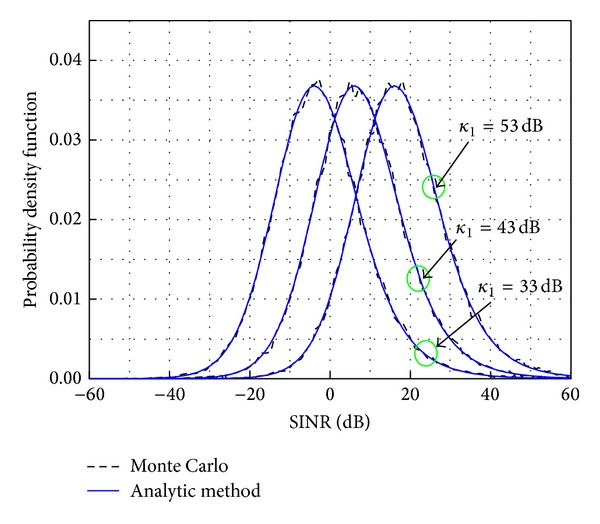
The pdf of the SINR for different values of ACIR, *κ*_*I*_.

Figure 4 shows three different plots depending on the ACIR value. The standard deviation of log-normal shadowing model *σ* is assumed to be 8 dB, and the cell radius, *R*_2_, is 50 m. As the ACIR, *κ*_*I*_, increases, the transmitted interfering power decreases. Therefore, the received signal power that is interfering with the other transmitter decreases, which leads to a higher SINR mean value.

The results presented in Figures 2–4 show that the analytical pdf is in agreement with the Monte Carlo simulation. Therefore, this analytical model could remove the need for Monte Carlo simulations when numerically calculating SINR.

The following is how to apply the derived analytical method for evaluating the interference effect of LTE's user equipment (UE) on the Trunked Radio Service (TRS)'s mobile station, using an adjacent frequency band. User equipment is any device used directly by an end-user to communicate. It can be a hand-held telephone, a laptop computer equipped with a mobile broadband adapter, or any other device [10]. We assume that LTE UE is mobile station like hand-held telephone.

**Figure 5:**
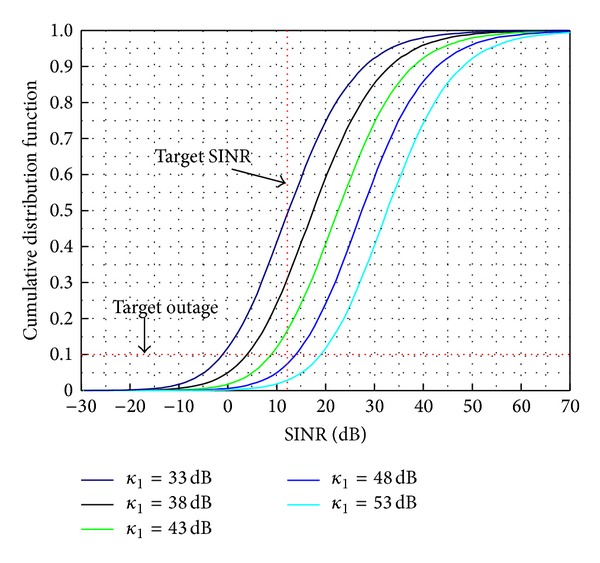
The cdf of the SINR for different values of ACIR, *κ*_*I*_.

Figure 5 presents the cumulative density function (cdf) of SINR for different values of ACIR at the TRS's mobile station. The cdf curve can be obtained from the pdf curve using an analytical method. The parameters in Table 1 are used for the simulation and the standard deviation *σ* of log-normal shadowing model is also assumed to be 8 dB and the hotspot cell radius *R*_2_ is assumed to be 200 m. The target SINR of TRS's mobile station is assumed to be 12 dB [11]. The target SINR means the reference value in which TRS's mobile station may be acceptable for some degradation in audio. We say that the service outage has occurred whenever SINR of the victim receiver is dropped below to the target SINR.

At the ACIR of *κ*_*I*_ = 33 dB, the probability to satisfy the target SINR is about 50% which corresponds to 50% occurrence in service outage, whereas *κ*_*I*_ = 53 dB gains a better outage probability of about 3%. If the acceptable TRS's outage probability is 10%, the ACIR value of about 46 dB can be proposed for coexistence between TRS's downlink and LTE's uplink. The requested ACIR value for coexistence increases, and it leads to a larger guard band.

## 4. Conclusion

This paper described the derivation of the pdf of SINR that is used for mobile-to-mobile interference in wireless networks without Monte Carlo simulations. The analytical method is compared with a Monte Carlo simulation for the verification of the derived pdf of SINR. We confirm that the analytical pdf is in agreement with the Monte Carlo simulations. The verified analytical pdf is used to evaluate the interference effect of LTE's user equipment on the TRS's mobile station using the adjacent frequency band. We confirm that the ACIR value of about 46 dB can be proposed for coexistence between TRS's downlink and LTE's uplink with the acceptable TRS outage probability being 10%. The proposed analytical method can be used to evaluate the interference effect between mobile stations. It can also be useful for providing technical criteria to result in an interference impact that is acceptable to both parties.

## Figures and Tables

**Table 1:** Parameters for the simulations.

Parameter	Value
Center frequency	900 MHz
Cell radius, *R*_1_	2000 m
Hotspot cell radius, *R*_2_	50 m
Path loss model	
BS-MS	Hata model
MS-MS	Motley-Keenan formula
Transmitted power of the desired base station	56 dBm
Transmitted power of the interfering mobile station	24 dBm
Adjacent channel interference ratio (ACIR)	33 dB
Monte Carlo runs	10,000
